# Supplementary material for: Effectiveness, Immunogenicity and Harms of Additional SARS-CoV-2 Vaccine Doses in Kidney Transplant Recipients: A Systematic Review
Source: Vaccines (Basel). 2023 Apr 18;11(4):863. doi: 10.3390/vaccines11040863 (PMC10141039; doi:10.3390/vaccines11040863)
Supplement: Supplementary file 1 [file vaccines-11-00863-s001.zip › vaccines-2264781-supplementary.pdf]

Content

Literature search 2

Cochrane Covid Study register (CCSR) 2

Web of Science (Core Collection) – Science Citation Index and Emerging Sources Citation Index 2

WHO COVID 19 Global literature on coronavirus disease 3

Risk of Bias 4

Supplemental Table S1: Risk of Bias in Overall Prognosis Studies (ROB-OPS) tool, Version 0.1 4

Changes to the protocol 5

Supplemental Figure S1: Risk of Bias Assessment 6

Supplemental Figure S2: Sensitivity analysis: Exclusion of all studies with high risk of bias 15

Supplemental Figure S3: Subgroup analysis: Immunosuppressive therapy 16

Supplemental Table S2: Main Study Characteristics 17

Supplemental Table S3: Antibody Titers 21

Supplemental Table S4: Neutralizing antibodies against Delta 23

Supplemental Table S5: Neutralizing antibodies against Omicron 24

Supplemental Table S6: T-cell response 25

Supplemental Table S7: Co-interventions 26

Supplemental Table S8: Transplant vintage of patients with and without seroconversion 27

Checklist of items to include when reporting a systematic review or meta-analysis 28

Literature search

Cochrane Covid Study register (CCSR)

kidney* or renal* or organ* or SOT
AND
transplant* or graft*
AND
vaccin* or biontech* or pfizer* or comirnaty* or cominarty* or corminaty* or BNT162* or "BNT 162" or "BNT 162b2" or tozinameran* or moderna* or spikevax* or 1273* or mRNA1273* or "TAK-919" or "CX-024414" or CX024414* or astrazeneca* or oxford* or vaxzevria* or AZD1222* or covishield* or ChAdOx* or Janssen* or "JNJ-78436735" or JNJ78436735* or VAC31518* or "VAC-31518" or "Johnson COVID-19" or "Johnson COVID19" or Ad26* or Ad5* or Sputnik* or rAd26* or rAd5* or gamaleya* or "Gam-COVID-Vac" or "recombinant adenovirus type" or "adenovirus vector" or "combined vector" or BBIBP* or sinopharm* or "vero cell" or "vero cells" or covilo or sinovac* or PiCoVac* or coronavac* or nuvaxovid* or novavax* or "NVX-CoV2373" or "SARS-CoV-2 rS" or "M1-Adjuvant" or "nanoparticle vaccine" or "Bharat biotech" or BBV152* or "BBV 152A" or "BBV 152B" or "BBV152C" or "BBV152D" or "BBV 152 A" or "BBV 152 B" or "BBV152 C" or "BBV152 D" or "BBV-152" or covaxin* or boost* or "third dose" or "third doses" or "third dosis" or "three dose" or "three doses" or "three dosis" or "fourth dose" or "fourth doses" or "fourth dosis" or "four dose" or "four doses" or "four dosis"

(no limitations set to study design)

Web of Science (Core Collection) – Science Citation Index and Emerging Sources Citation Index

- Science Citation Index Expanded (1945-present)
- Emerging Sources Citation Index (2015-present)
- Limit to 2020 - 2022

#1 (TI=(COVID OR COVID19 OR "SARS-CoV-2" OR "SARS-CoV2" OR SARSCoV2 OR "SARSCoV-2" OR "SARS coronavirus 2" OR "2019 nCoV" OR "2019nCoV" OR "2019-novel CoV" OR "nCov 2019" OR "nCov 19" OR "severe acute respiratory syndrome coronavirus 2" OR "novel coronavirus disease" OR "novel corona virus disease" OR "corona virus disease 2019" OR "coronavirus disease 2019" OR "novel coronavirus pneumonia" OR "novel corona virus pneumonia" OR "severe acute respiratory syndrome coronavirus 2")) OR AB=(COVID OR COVID19 OR "SARS-CoV-2" OR "SARS-CoV2" OR SARSCoV2 OR "SARSCoV-2" OR "SARS coronavirus 2" OR "2019 nCoV" OR "2019nCoV" OR "2019-novel CoV" OR "nCov 2019" OR "nCov 19" OR "severe acute respiratory syndrome coronavirus 2" OR "novel coronavirus disease" OR "novel corona virus disease" OR "corona virus disease 2019" OR "coronavirus disease 2019" OR "novel coronavirus pneumonia" OR "novel corona virus pneumonia" OR "severe acute respiratory syndrome coronavirus 2")
#2 (TI=(vaccin* OR biontech* OR pfizer* OR comirnaty* OR cominarty* OR corminaty* OR BNT162* OR "BNT 162" OR "bnt 162b2" OR tozinameran* OR moderna* OR spikevax* OR 1273* OR mRNA1273* OR "TAK-919" OR "CX-024414" OR CX024414* OR astrazeneca* OR oxford* OR

vaxzevria* OR AZD1222* OR covishield* OR ChAdOx* OR Janssen* OR "JNJ-78436735" OR JNJ78436735* OR VAC31518* OR "VAC-31518" OR "Johnson COVID-19" OR "Johnson COVID19" OR Ad26* OR Ad5* OR Sputnik* OR rAd26* OR rAd5* OR gamaleya* OR "Gam-COVID-Vac" OR "recombinant adenovirus type" OR "adenovirus vector" OR "combined vector" OR BBIBP* OR sinopharm* OR "vero cell" OR "vero cells" OR covilo OR sinovac* OR PiCoVac* OR coronavac* OR Nuvaxovid* OR Novavax* OR "NVX-CoV2373" OR "SARS-CoV-2 rS" OR "M1-Adjuvant" OR "nanoparticle vaccine" OR "Bharat biotech" OR BBV152* OR "BBV 152A" OR "BBV 152B" OR "BBV152C" OR "BBV152D" OR "BBV 152 A" OR "BBV 152 B" OR "BBV152 C" OR "BBV152 D" OR "BBV-152" OR Covaxin* OR boost* OR "third dose" OR "third doses" OR "third dosis" OR "three dose" OR "three doses" OR "three dosis")) OR AB=(vaccin* OR biontech* OR pfizer* OR comirnaty* OR cominarty* OR corminaty* OR BNT162* OR "BNT 162" OR "bnt 162b2" OR tozinameran* OR moderna* OR spikevax* OR 1273* OR mRNA1273* OR "TAK-919" OR "CX-024414" OR CX024414* OR astrazeneca* OR oxford* OR vaxzevria* OR AZD1222* OR covishield* OR ChAdOx* OR Janssen* OR "JNJ-78436735" OR JNJ78436735* OR VAC31518* OR "VAC-31518" OR "Johnson COVID-19" OR "Johnson COVID19" OR Ad26* OR Ad5* OR Sputnik* OR rAd26* OR rAd5* OR gamaleya* OR "Gam-COVID-Vac" OR "recombinant adenovirus type" OR "adenovirus vector" OR "combined vector" OR BBIBP* OR sinopharm* OR "vero cell" OR "vero cells" OR covilo OR sinovac* OR PiCoVac* OR coronavac* OR Nuvaxovid* OR Novavax* OR "NVX-CoV2373" OR "SARS-CoV-2 rS" OR "M1-Adjuvant" OR "nanoparticle vaccine" OR "Bharat biotech" OR BBV152* OR "BBV 152A" OR "BBV 152B" OR "BBV152C" OR "BBV152D" OR "BBV 152 A" OR "BBV 152 B" OR "BBV152 C" OR "BBV152 D" OR "BBV-152" OR Covaxin* OR boost* OR "third dose" OR "third doses" OR "third dosis" OR "three dose" OR "three doses" OR "three dosis" OR "fourth dose" OR "fourth doses" OR "fourth dosis" OR "four dose" OR "four doses" OR "four dosis")
#3 (TI=(kidney* OR renal* OR organ* OR SOT)) OR AB=(kidney* OR renal* OR organ* OR SOT)
#4 (TI=(transplant* or graft*)) OR AB=(transplant* or graft*)
#5 #1 AND #2 AND #3 AND #4

WHO COVID 19 Global literature on coronavirus disease

(kidney* OR renal* OR organ* OR sot)
AND
(transplant* OR graft*)
AND
(vaccin* OR biontech* OR pfizer* OR comirnaty* OR cominarty* OR corminaty* OR bnt162* OR "BNT 162" OR "bnt 162b2" OR tozinameran* OR moderna* OR spikevax* OR 1273* OR mrna1273* OR "TAK-919" OR "CX-024414" OR cx024414* OR astrazeneca* OR oxford* OR vaxzevria* OR azd1222* OR covishield* OR chadox* OR janssen* OR "JNJ-78436735" OR jnj78436735* OR vac31518* OR "VAC-31518" OR "Johnson COVID-19" OR "Johnson COVID19" OR ad26* OR ad5* OR sputnik* OR rad26* OR rad5* OR gamaleya* OR "Gam-COVID-Vac" OR "recombinant adenovirus type" OR "adenovirus vector" OR "combined vector" OR bbibp* OR sinopharm* OR "vero cell" OR "vero cells" OR covilo OR sinovac* OR picovac* OR coronavac* OR nuvaxovid* OR novavax* OR "NVX-CoV2373" OR "SARS-CoV-2 rS" OR "M1-Adjuvant" OR "nanoparticle vaccine" OR "Bharat biotech" OR bbv152* OR "BBV 152A" OR "BBV 152B" OR "BBV152C" OR "BBV152D" OR "BBV 152 A" OR "BBV 152 B" OR "BBV152 C" OR "BBV152 D" OR "BBV-152" OR covaxin* OR boost* OR "third dose" OR "third doses" OR "third dosis" OR OR "fourth dose" OR "fourth doses" OR "fourth dosis" OR "four dose" OR "four doses" OR "four dosis")

Risk of Bias

Our research question comprised 1) the overall outcome for kidney transplant recipients and 2) the comparative effectiveness of COVID-19 vaccines. We therefore used a tool which is currently in its first phase of development by members of the Cochrane Prognosis Methods Group and Cochrane Haematology, the Risk of Bias in Overall Prognosis Studies (ROB-OPS) tool for 1) and the ROBINS-I tool for non-randomized studies of interventions and the ROB-2 tool for 2).

We assessed the risk of bias by study and by outcome. Each domain received an overall rating of either low, moderate or high rating for risk of bias. Based on the domain-rating for each study and outcome, an overall rating for the outcome per study was created as follows:

- Low risk of bias: no domain rated as high or moderate risk of bias per study and outcome
- Moderate risk of bias: at least one rating as moderate risk of bias per study and outcome group.
- High risk of bias: at least one domain rated as high risk of bias per study and outcome.

Details on the RoB-OPS-tool are to be found at: https://osf.io/dfk2r. In Supplemental Table1, the signaling questions and domains can be found

Each domain contains one or more signalling questions and rating options to guide to either “yes”, “probably yes”, “probably no”, “no” or “not sufficient information”.

Supplemental Table S1: Risk of Bias in Overall Prognosis Studies (ROB-OPS) tool, Version 0.1

Domain	Signalling question
1. Participants	1.1 Was the method of participant recruitment appropriate?
	1.2 Were all inclusions and exclusions appropriate?
2. Outcome	2.1 Was the outcome of interest defined appropriately?

	2.2 Was the used outcome measure appropriate?
	2.3 Was the duration of follow-up appropriate?
	2.4 Was the outcome determined without knowledge of relevant baseline characteristics?
3. Analysis	3.1 Were outcome data available for all included participants or likely to be unrelated to the outcome value?
	3.2 Were missing outcome data handled appropriately?
	3.3 Was the statistical method for estimating the overall prognosis appropriate?
	3.4 Were complexities in the analysed data handled appropriately?
4. selective reporting	4.1 Is the reported outcome likely to be unselected?

Changes to the protocol

Comparisons
We aimed to compare seroconversion in patients receiving mRNA-1273 vs. BNT162b2. We found only two studies reporting on this outcome. We decided not to create descriptive summary of finding- tables due to the large number of endpoints and tables we provide in our review.

Outcome measures
Due to the very low number of studies reporting on time to infection, we decided that evidence could not reasonably be gathered from the actual information and do not report it in the review. The same applies to the composite endpoint admission to hospital or death and the endpoint death. we decided not to report mild side effects of vaccination as this has already been published several times and we did not see any additional benefit of this endpoint.
We did not predefine our reporting on correlates of protection. We assumed that several studies would report neutralization specific antibodies, but this was not the case. But we did find that studies reported on certain antibody levels associated with protection against infection. This information was extracted for all studies included in our review.

Supplemental Figure S1: Risk of Bias Assessment

Seroconversion: RoB-OPS

Risk of bias

Study	D1	D2	D3	D4	Overall
COVIReD 2021	-	X	X	+	X
Massa 2021	+	+	+	+	+
Benotmane 2021	-	+	+	+	-
Noble 2021	-	-	+	+	-
Roch 2022	-	+	+	+	-
DiaVacc 2021	+	+	+	+	+
Kantauskaite 2022	+	+	+	+	+
Schrezenmeier 2022	-	+	+	+	-
McEvoy 2022	-	+	+	+	-
Abravanel 2022	-	+	+	+	-
Tauzin 2022	-	+	X	+	X
COVAC-Tx 2022	+	+	X	+	X
Benning 2022	+	+	+	+	+
Bruminhent 2022	+	+	+	+	+
Caglioti 2022	+	+	+	+	+
Cassaniti 2022	-	+	+	+	-
Schimpf 2022	-	+	+	?	-
Midtvedt 2022	-	+	+	+	-
Mitchell 2022	-	-	+	?	-
Grupper 2022	-	X	+	+	X
Yahav 2022	+	+	+	+	+

D1: Participants
D2: Outcomes
D3: Analysis
D4: Selective Reporting

Judgement
X High
- Moderate
+ Low
? No information

Seroconversion: ROBINS-I

Risk of bias domains

Study	D1	D2	D3	D4	D5	D6	D7	Overall
Caillard 2022	-	+	+	+	+	+	+	-
Schrezenmeier 2021	-	+	+	+	+	+	+	-
COVINEPH 2021	-	+	+	+	+	+	+	-

Seroconversion: ROB2

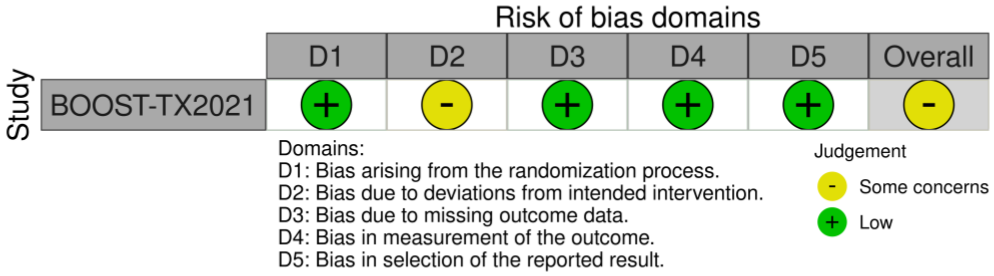

Neutralization against Delta or Omicron VOC: RoB-OPS

Risk of bias

Study	D1	D2	D3	D4	Overall
Al Jurdi 2022	-	+	+	+	-
Massa 2021	+	+	+	+	+
Roch 2022	-	+	+	+	-
Kantauskaite 2022	+	+	+	+	+
Benotmane 2022	-	+	+	+	-
McEvoy 2022	-	+	+	+	-
Tauzin 2022	-	+	X	+	X
Benning 2022	+	+	+	+	+
Kumar 2022	+	+	+	+	+

T-cell response: RoB-OPS

Risk of bias

Study	D1	D2	D3	D4	Overall
DiaVacc 2021	+	+	X	X	X
Bruminhent 2022	+	+	+	+	+
Cassaniti 2022	-	+	+	+	-
Yahav 2022	+	+	+	+	+

T-cell response: ROBINS-I

Risk of bias

Study	D1	D2	D3	D4	D5	D6	D7	Overall
Thomson 2022	-	+	+	+	+	+	-	-

T-cell response: ROB2

Risk of bias domains

Study	D1	D2	D3	D4	D5	Overall
BOOST-TX2021	+	-	+	+	+	-

T-cell response: ROB2
Efficacy (Any infection): RoB-OPS

Efficacy (Any infection): RoB-OPS

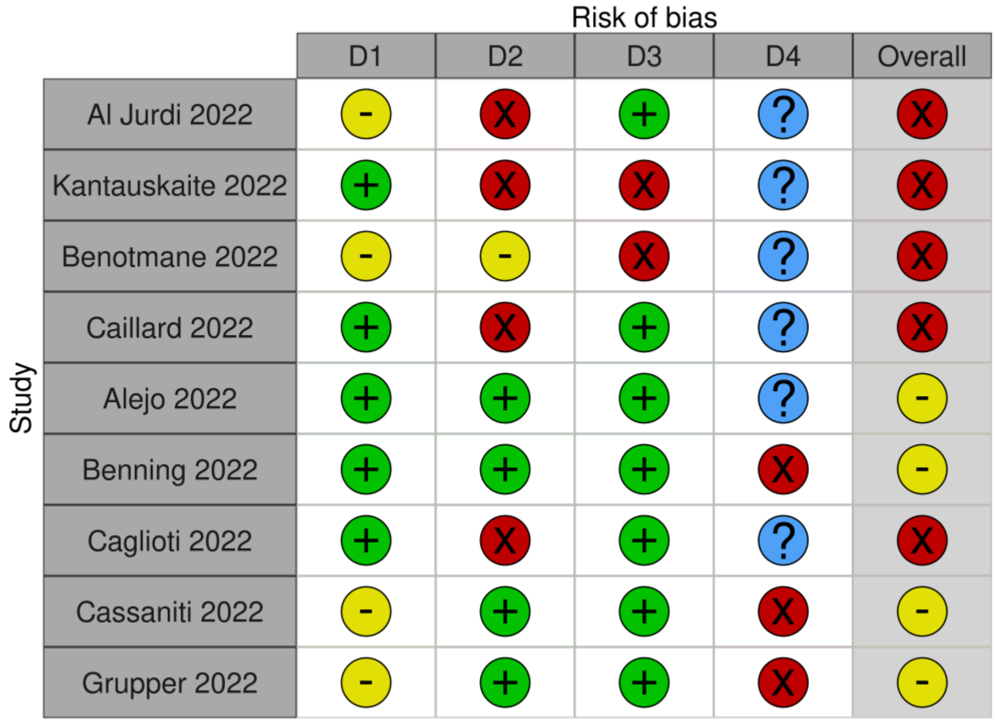

Efficacy (Any infection): ROBINS-I

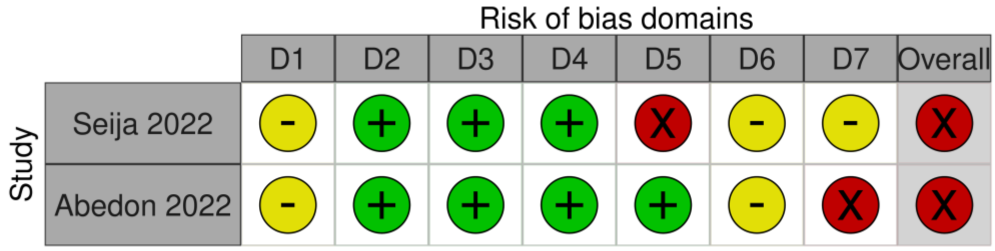

Efficacy (Any infection): ROB2

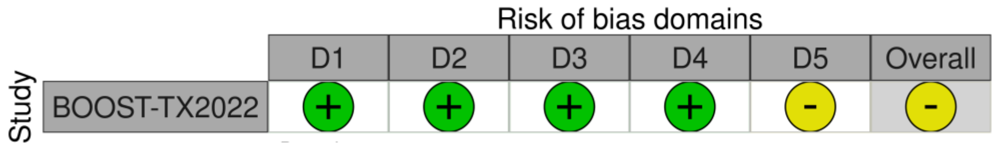

Efficacy (Hospitalization and Death): ROB-OPS

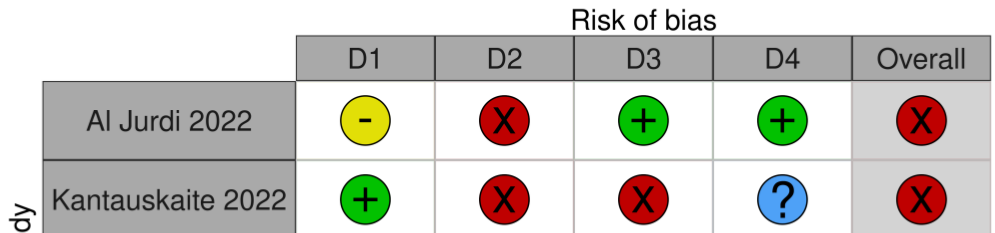

Efficacy (Hospitalization and Death): ROBINS-I

Study	D1	D2	D3	D4	D5	D6	D7	Overall
Seija 2022	-	+	+	+	X	-	-	X

Efficacy (Hospitalization and Death): ROB2

Study	D1	D2	D3	D4	D5	Overall
BOOST-TX2022	+	+	+	+	-	-

Safety (De-novo DSA): RoB-OPS

Study	D1	D2	D3	D4	Overall
Al Jurdi 2022	-	+	+	+	-
Massa 2021	+	+	+	+	+
Kantauskaite 2022	+	+	+	+	+
Schrezenmeier 2022	-	+	+	+	-
Cassaniti 2022	-	+	+	+	-

Safety (De-novo DSA): ROBINS-I

Study	D1	D2	D3	D4	D5	D6	D7	Overall
Regele 2022	X	+	!	+	!	!	!	!

Safety (Acute graft rejection): RoB-OPS

Study	D1	D2	D3	D4	Overall
	Risk of bias				
Al Jurdi 2022	-	+	+	+	-
Massa 2021	+	+	+	+	+
Benotmane 2022	-	-	+	+	-
Schrezenmeier 2022	-	+	+	+	-
Bruminhent 2022	+	X	+	+	X
Cassaniti 2022	-	X	+	+	X
Grupper 2022	-	+	+	+	-
Yahav 2022	+	+	+	+	+
Midtvedt 2022	-	X	+	+	X

Safety (Acute graft rejection): ROBINS-I

Study	D1	D2	D3	D4	D5	D6	D7	Overall
	Risk of bias domains							
Regele 2022	X	+	!	+	!	!	!	!
Seija 2022	-	+	+	+	X	+	-	X

Safety (Serious adverse events): RoB-OPS

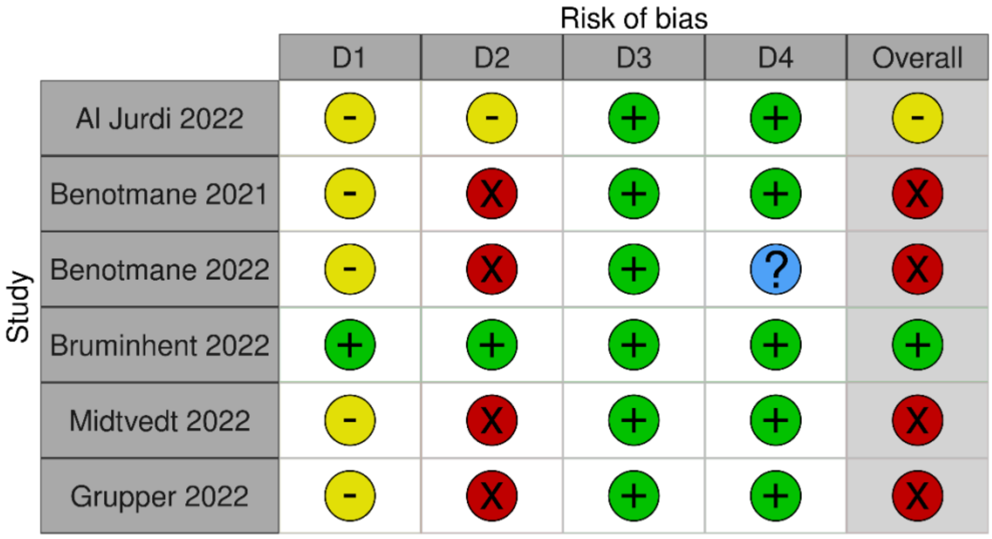

Safety (Serious adverse events): ROBINS-I

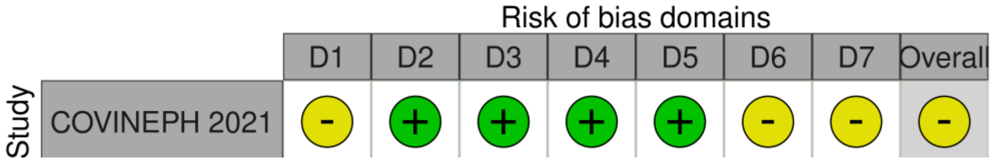

Safety (Serious adverse events): ROB2

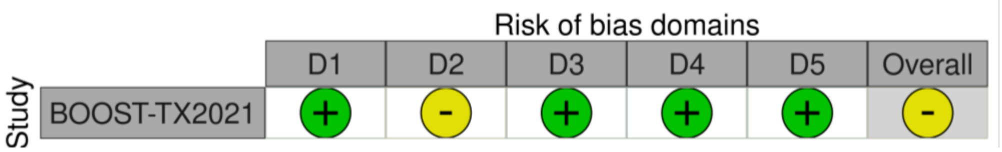

Supplemental Figure S2 Sensitivity analysis: Exclusion of all studies with high risk of bias.

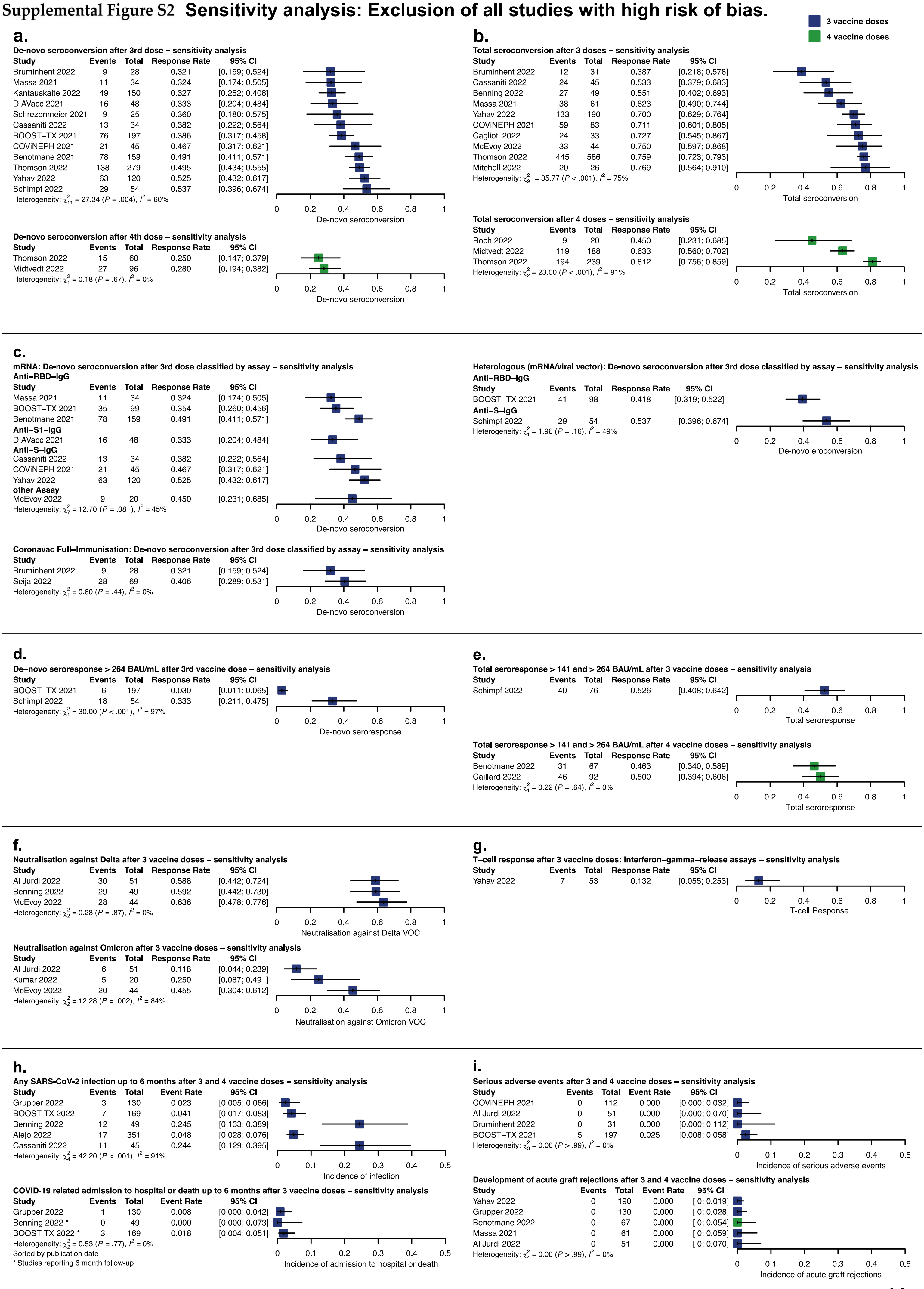

a. De-novo seroconversion after 3rd dose – sensitivity analysis

Study	Events	Total	Response Rate	95% CI
Bruminhent 2022	9	28	0.321	[0.159; 0.524]
Massa 2021	11	34	0.324	[0.174; 0.505]
Kantauskaite 2022	49	150	0.327	[0.252; 0.408]
DIAVacc 2021	16	48	0.333	[0.204; 0.484]
Schrezenmeier 2021	9	25	0.360	[0.180; 0.575]
Cassaniti 2022	13	34	0.382	[0.222; 0.564]
BOOST–TX 2021	76	197	0.386	[0.317; 0.458]
COViNEPH 2021	21	45	0.467	[0.317; 0.621]
Benotmane 2021	78	159	0.491	[0.411; 0.571]
Thomson 2022	138	279	0.495	[0.434; 0.555]
Yahav 2022	63	120	0.525	[0.432; 0.617]
Schimpf 2022	29	54	0.537	[0.396; 0.674]

Heterogeneity: $\chi^2_{11} = 27.34$ ($P = .004$), $I^2 = 60\%$

De-novo seroconversion

De-novo seroconversion after 4th dose – sensitivity analysis

Study	Events	Total	Response Rate	95% CI
Thomson 2022	15	60	0.250	[0.147; 0.379]
Midtvedt 2022	27	96	0.280	[0.194; 0.382]

Heterogeneity: $\chi^2_1 = 0.18$ ($P = .67$), $I^2 = 0\%$

De-novo seroconversion

b. Total seroconversion after 3 doses – sensitivity analysis

Study	Events	Total	Response Rate	95% CI
Bruminhent 2022	12	31	0.387	[0.218; 0.578]
Cassaniti 2022	24	45	0.533	[0.379; 0.683]
Benning 2022	27	49	0.551	[0.402; 0.693]
Massa 2021	38	61	0.623	[0.490; 0.744]
Yahav 2022	133	190	0.700	[0.629; 0.764]
COViNEPH 2021	59	83	0.711	[0.601; 0.805]
Caglioti 2022	24	33	0.727	[0.545; 0.867]
McEvoy 2022	33	44	0.750	[0.597; 0.868]
Thomson 2022	445	586	0.759	[0.723; 0.793]
Mitchell 2022	20	26	0.769	[0.564; 0.910]

Heterogeneity: $\chi^2_9 = 35.77$ ($P < .001$), $I^2 = 75\%$

Total seroconversion

Total seroconversion after 4 doses – sensitivity analysis

Study	Events	Total	Response Rate	95% CI
Roch 2022	9	20	0.450	[0.231; 0.685]
Midtvedt 2022	119	188	0.633	[0.560; 0.702]
Thomson 2022	194	239	0.812	[0.756; 0.859]

Heterogeneity: $\chi^2_2 = 23.00$ ($P < .001$), $I^2 = 91\%$

Total seroconversion

c. mRNA: De-novo seroconversion after 3rd dose classified by assay – sensitivity analysis

Study	Events	Total	Response Rate	95% CI
**Anti–RBD–IgG**				
Massa 2021	11	34	0.324	[0.174; 0.505]
BOOST–TX 2021	35	99	0.354	[0.260; 0.456]
Benotmane 2021	78	159	0.491	[0.411; 0.571]
**Anti–S1–IgG**				
DIAVacc 2021	16	48	0.333	[0.204; 0.484]
**Anti–S–IgG**				
Cassaniti 2022	13	34	0.382	[0.222; 0.564]
COViNEPH 2021	21	45	0.467	[0.317; 0.621]
Yahav 2022	63	120	0.525	[0.432; 0.617]
**other Assay**				
McEvoy 2022	9	20	0.450	[0.231; 0.685]

Heterogeneity: $\chi^2_7 = 12.70$ ($P = .08$), $I^2 = 45\%$

De-novo seroconversion

Heterologous (mRNA/viral vector): De-novo seroconversion after 3rd dose classified by assay – sensitivity analysis

Study	Events	Total	Response Rate	95% CI
**Anti–RBD–IgG**				
BOOST–TX 2021	41	98	0.418	[0.319; 0.522]
**Anti–S–IgG**				
Schimpf 2022	29	54	0.537	[0.396; 0.674]

Heterogeneity: $\chi^2_1 = 1.96$ ($P = .16$), $I^2 = 49\%$

De-novo eroconversion

Coronavac Full–Immunisation: De-novo seroconversion after 3rd dose classified by assay – sensitivity analysis

Study	Events	Total	Response Rate	95% CI
Bruminhent 2022	9	28	0.321	[0.159; 0.524]
Seija 2022	28	69	0.406	[0.289; 0.531]

Heterogeneity: $\chi^2_1 = 0.60$ ($P = .44$), $I^2 = 0\%$

De-novo seroconversion

d. De–novo seroresponse > 264 BAU/mL after 3rd vaccine dose – sensitivity analysis

Study	Events	Total	Response Rate	95% CI
BOOST–TX 2021	6	197	0.030	[0.011; 0.065]
Schimpf 2022	18	54	0.333	[0.211; 0.475]

Heterogeneity: $\chi^2_1 = 30.00$ ($P < .001$), $I^2 = 97\%$

De-novo seroresponse

e. Total seroresponse > 141 and > 264 BAU/mL after 3 vaccine doses – sensitivity analysis

Study	Events	Total	Response Rate	95% CI
Schimpf 2022	40	76	0.526	[0.408; 0.642]

Total seroresponse

Total seroresponse > 141 and > 264 BAU/mL after 4 vaccine doses – sensitivity analysis

Study	Events	Total	Response Rate	95% CI
Benotmane 2022	31	67	0.463	[0.340; 0.589]
Caillard 2022	46	92	0.500	[0.394; 0.606]

Heterogeneity: $\chi^2_1 = 0.22$ ($P = .64$), $I^2 = 0\%$

Total seroresponse

f. Neutralisation against Delta after 3 vaccine doses – sensitivity analysis

Study	Events	Total	Response Rate	95% CI
Al Jurdi 2022	30	51	0.588	[0.442; 0.724]
Benning 2022	29	49	0.592	[0.442; 0.730]
McEvoy 2022	28	44	0.636	[0.478; 0.776]

Heterogeneity: $\chi^2_2 = 0.28$ ($P = .87$), $I^2 = 0\%$

Neutralisation against Delta VOC

Neutralisation against Omicron after 3 vaccine doses – sensitivity analysis

Study	Events	Total	Response Rate	95% CI
Al Jurdi 2022	6	51	0.118	[0.044; 0.239]
Kumar 2022	5	20	0.250	[0.087; 0.491]
McEvoy 2022	20	44	0.455	[0.304; 0.612]

Heterogeneity: $\chi^2_2 = 12.28$ ($P = .002$), $I^2 = 84\%$

Neutralisation against Omicron VOC

g. T–cell response after 3 vaccine doses: Interferon–gamma–release assays – sensitivity analysis

Study	Events	Total	Response Rate	95% CI
Yahav 2022	7	53	0.132	[0.055; 0.253]

T-cell Response

h. Any SARS-CoV-2 infection up to 6 months after 3 and 4 vaccine doses – sensitivity analysis

Study	Events	Total	Event Rate	95% CI
Grupper 2022	3	130	0.023	[0.005; 0.066]
BOOST TX 2022	7	169	0.041	[0.017; 0.083]
Benning 2022	12	49	0.245	[0.133; 0.389]
Alejo 2022	17	351	0.048	[0.028; 0.076]
Cassaniti 2022	11	45	0.244	[0.129; 0.395]

Heterogeneity: $\chi^2_4 = 42.20$ ($P < .001$), $I^2 = 91\%$

Incidence of infection

COVID-19 related admission to hospital or death up to 6 months after 3 vaccine doses – sensitivity analysis

Study	Events	Total	Event Rate	95% CI
Grupper 2022	1	130	0.008	[0.000; 0.042]
Benning 2022 *	0	49	0.000	[0.000; 0.073]
BOOST TX 2022 *	3	169	0.018	[0.004; 0.051]

Heterogeneity: $\chi^2_2 = 0.53$ ($P = .77$), $I^2 = 0\%$

Sorted by publication date

* Studies reporting 6 month follow-up

Incidence of admission to hospital or death

i. Serious adverse events after 3 and 4 vaccine doses – sensitivity analysis

Study	Events	Total	Event Rate	95% CI
COViNEPH 2021	0	112	0.000	[0.000; 0.032]
Al Jurdi 2022	0	51	0.000	[0.000; 0.070]
Bruminhent 2022	0	31	0.000	[0.000; 0.112]
BOOST–TX 2021	5	197	0.025	[0.008; 0.058]

Heterogeneity: $\chi^2_3 = 0.00$ ($P > .99$), $I^2 = 0\%$

Incidence of serious adverse events

Development of acute graft rejections after 3 and 4 vaccine doses – sensitivity analysis

Study	Events	Total	Event Rate	95% CI
Yahav 2022	0	190	0.000	[0; 0.019]
Grupper 2022	0	130	0.000	[0; 0.028]
Benotmane 2022	0	67	0.000	[0; 0.054]
Massa 2021	0	61	0.000	[0; 0.059]
Al Jurdi 2022	0	51	0.000	[0; 0.070]

Heterogeneity: $\chi^2_4 = 0.00$ ($P > .99$), $I^2 = 0\%$

Incidence of acute graft rejections

Supplemental Figure S3 Subgroup analysis: Immunosuppressive therapy.

3 vaccine doses
4 vaccine doses

De-novo seroconversion after 3rd dose in regimens including Belatacept

Study	Events	Total	Response Rate	95% CI
Noble 2021	5	41	0.122	[0.041; 0.262]
Abravanel 2022	11	62	0.177	[0.092; 0.295]
Mitchell 2022	4	20	0.200	[0.057; 0.437]

Heterogeneity: $\chi^2_2 = 0.79$ ($P = .67$), $I^2 = 0\%$

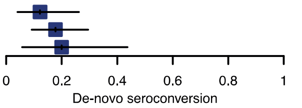

De-novo seroconversion after 3rd dose in regimens including MMF

Study	Events	Total	Response Rate	95% CI
Kantauskaite 2022	53	164	0.323	[0.252; 0.401]
Bruminhent 2022	11	29	0.379	[0.207; 0.577]
Seija 2022	28	70	0.400	[0.285; 0.524]

Heterogeneity: $\chi^2_2 = 1.39$ ($P = .50$), $I^2 = 0\%$

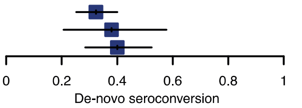

De-novo seroconversion after 4th dose in regimens including MMF

Study	Events	Total	Response Rate	95% CI
Seija 2022	13	37	0.351	[0.202; 0.525]

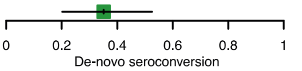

De-novo seroconversion after 3rd dose in regimens with triple immunosuppression

Study	Events	Total	Response Rate	95% CI
Kantauskaite 2022	50	162	0.309	[0.239; 0.386]
Seija 2022	22	59	0.373	[0.250; 0.509]
Thomson 2022	57	99	0.576	[0.472; 0.675]

Heterogeneity: $\chi^2_2 = 17.92$ ($P < .001$), $I^2 = 89\%$

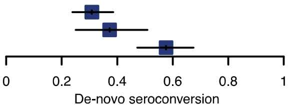

De-novo seroconversion after 4th dose in regimens with triple immunosuppression

Study	Events	Total	Response Rate	95% CI
Seija 2022	11	33	0.333	[0.180; 0.518]
Thomson 2022	20	41	0.488	[0.329; 0.649]

Heterogeneity: $\chi^2_1 = 1.77$ ($P = .18$), $I^2 = 44\%$

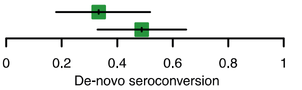

De-novo seroconversion after 3rd dose in regimens including steroids

Study	Events	Total	Response Rate	95% CI
Kantauskaite 2022	51	164	0.311	[0.241; 0.388]
Seija 2022	32	73	0.438	[0.322; 0.559]

Heterogeneity: $\chi^2_1 = 3.57$ ($P = .06$), $I^2 = 72\%$

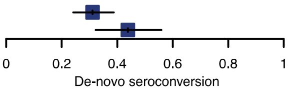

De-novo seroconversion after 3rd dose in regimens including CNI

Study	Events	Total	Response Rate	95% CI
Kantauskaite 2022	56	167	0.335	[0.264; 0.412]
Bruminhent 2022	11	29	0.379	[0.207; 0.577]

Heterogeneity: $\chi^2_1 = 0.21$ ($P = .65$), $I^2 = 0\%$

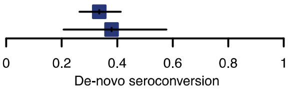

De-novo seroconversion after 3rd dose in regimens with dual immunosuppression

Study	Events	Total	Response Rate	95% CI
Kantauskaite 2022	6	12	0.500	[0.211; 0.789]
Thomson 2022	142	209	0.679	[0.612; 0.742]

Heterogeneity: $\chi^2_1 = 1.59$ ($P = .21$), $I^2 = 37\%$

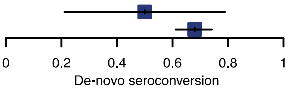

De-novo seroconversion after 4th dose in regimens with dual immunosuppression

Study	Events	Total	Response Rate	95% CI
Thomson 2022	74	88	0.841	[0.748; 0.91]

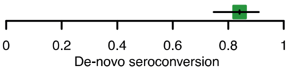

Supplemental Table S2: Main Study Characteristics

	Study information					Population Characteristics						Information on Vaccination						
Study	Country	Study design	Participants	Special Population	Intervention	Study population	Age	*N* female (%)	Transplant vintage (months)*	Triple immuno-suppressive therapy (%)	*N* with Previous SARS-CoV-2 Infection n (%)	Primary vaccination series	Third dose	Fourth dose	Total vaccine doses	Last vaccination interval (days)*	Follow up	*N* receiving additional dose
Abedon 2022	USA	case series	KTRs + 4 KPTRs+1KLTRs	no	BNT126b2, mRNA-1273, Ad26.COV2.S **separately reported**	including seropositive patients	54 (45-71)	22 (69)	91.2 (43.2-156)	NR	0	mRNA	Ad26.COV2.S or BNT162b2 or mRNA-1273	none	3	NR	6 months	32
Abravanel 2022	France	prospective cohort study	SOTRs (68 in total, 62 KTRs)	Belatacept treated patients	BNT162b2	including seropositive patients	NR	NR	NR	NR	0	BNT162b2	BNT162b2	none	3	56 (11)	1 month	62
Al Jurdi 2022	USA	prospective cohort study	KTRs	no	BNT162b2, mRNA-1273	including seropositive patients	63 (54-69)	23 (43)	43 (4-443)	NR	4 (8)	mRNA	mRNA	none	3	187 (181-193)	1 month	51
Alejo 2022	USA	prospective cohort study	SOTRs (660 in total, 351 KTRs)	no	BNT162b2, mRNA-1273, AD.26.COV2.S	including seropositive patients	NR	NR	NR	NR	0	Ad26.COV2.S or BNT162b2 or mRNA-1273	Ad26.COV2.S or BNT162b2 or mRNA-1273	none	≥3	NR	3 months	351
Benning 2022	Germany	prospective cohort study	KTRs	no	mRNA	including seropositive patients	55 (46-65)	20 (41)	97.2 (28.8-163.2)	>80%	0	mRNA or ChAdOx1	mRNA	none	3	138 (117-162)	3 weeks	49
Benotmane 2021	France	prospective cohort study	KTRs	no	mRNA-1273	including only patients without seroconversion after primary series	57.6 (49.6-66.1)	61 (38.4)	63.6 (22.8-133.12)	50 %	0	mRNA-1273	mRNA-1273	none	3	51 (48-59)	1 month	159
Benotmane 2022	France	prospective cohort study	KTRs	no	mRNA-1273	including seropositive patients	56.6 (47-64.4)	26 (39)	73.2 (26.4-136.8)	>75%	0	mRNA-1273	mRNA-1273	mRNA-1273	4	68 (63-82)	1 month	67
BOOST-TX 2021	Austria	RCT	KTRs	no	BNT162b2, mRNA-1273, Ad26.COV2.S **separately reported**	including only patients without seroconversion after primary series	61.2 (12.4)	82 (42)	56.16	90 %	0	mRNA	Ad26.COV2.S or mRNA	none	3	78 (56-87)	1 month	197
Bruminhent 2022	Thailand	prospective cohort study	KTRs, 28 PD, and 31 HD patients	no	ChAdOx1-S	including seropositive patients	51 (42-54)	13 (42)	NR	NR	0	Coronavac	ChAdOx1-S	none	3	30-60	4-6 weeks	31
Caglioti 2022	Italy	prospective cohort study	KTRs	no	BNT162b2	including seropositive patients	55.4 (12.8)	14 (42)	NR	61 %	NR	BNT162b2	BNT162b2	none	3	180	3 weeks	33

	Study information					Population Characteristics						Information on Vaccination						
Study	Country	Study design	Participants	Special Population	Intervention	Study population	Age	*N* female (%)	Transplant vintage (months)*	Triple immuno-suppressive therapy (%)	*N* with Previous SARS-CoV-2 Infection n (%)	Primary vaccination series	Third dose	Fourth dose	Total vaccine doses	Last vaccination interval (days)*	Follow up	*N* receiving additional dose
Caillard 2022	France	case series	KTRs	no	BNT162b2, mRNA-1273, **separately reported**	including only patients with low or no seroconversion after primary series	55.9 (47.1-64.2)	28 (30.4)	66 (27.6-136.8)	70 %	NR	mRNA	mRNA	mRNA	4	68 (61-74.7)	1 month	92
Cassaniti 2022	Italy	prospective cohort study	KTRs	no	BNT162b2	including seropositive patients	52.6 (47.2-60)	22 (48.9)	51 (26-78.5)	64 %	0	BNT162b2	BNT162b 2	none	3	180	3 weeks	45
COVAC-Tx 2022	Denmark	prospective cohort study	SOTRs (281 KTRs)	no	BNT162b2	including seropositive patients	NR	NR	NR	NR	n.a.	BNT162b2	BNT162b 2	none	3	NR	5-6 weeks	240
COViNEPH 2021	Poland	prospective cohort study	KTRs	no	BNT162b2, mRNA-1273, **separately reported**	including seropositive patients	55 (42-63)	29 (34.94)	96 (42-180)	70 %	0	mRNA	mRNA	none	3	90	2-3 weeks	112
COVIReD 2021	Israel	prospective cohort study	KTRs	no	BNT162b2	including seropositive patients	NR	NR	NR	NR	0	BNT162b2	BNT162b 2	none	3	NR	8-117 days	118
de Boer 2022	The Netherlands	RCT	KTRs	elderly KTRs recently receiving transplant	mRNA vaccine + co-intervention: MPA reduction vs. MPA free triple immunosuppressive regimen	including seropositive patients	72(4)	12 (38)	7 (5-11.24) or 8.5 (4.5-10.5)	100 %	0	mRNA	mRNA	none	3	202 (184-215)	2 months	22
DIAVacc 2021	Germany	prospective cohort study	KTRs	no	BNT162b2	including seropositive patients	57 (14.4)	18(37)	90±72	<50%	0	BNT162b2	BNT162b 2	none	3	68± 1	1 month	48
Grupper 2022	Israel	prospective cohort study	KTRs, 4 LKTRs, 8 PKTRs	no	BNT162b2	including seropositive patients	58 (12.8)	29 (60.4)	66±88.1	77.60 %	0	BNT162b2	BNT162b 2	none	3	150	3 weeks	130
Kantauskaite 2022	Germany	prospective cohort study	KTRs	no	BNT162b2, ChadOx1, Ad26.COV2.S **not reported separately**	including only patients without seroconversion after primary series	59.4	60 (34.5)	46 (22-111) for seronegative vs. 92 (56-152) for seropositive	88 %	0	mRNA	Ad26.COV2.S or ChadOx1 or BNT162b 2	none	3	76±25	3 weeks	174
Kumar 2022	Canada	prospective cohort study	SOTRs, KTRs	no	mRNA-1273	including seropositive patients	NR	NR	NR	NR	0	mRNA-1273	mRNA-1273	none	3	90	1 month	20
Malahe 2022	The Netherlands	prospective cohort study	SOTRs, KTRs	no	mRNA	including seropositive patients	54 (range: 34-84)	19 (44)	56% >5 years, 7% < 1y, 12% 1-5y, 24% >5y	<50%	NR	mRNA	mRNA	none	3	NR	2 months	38
Massa 2021	France	prospective cohort study	KTRs	no	BNT162b2	including seropositive patients	58.0 (47.1-66)	17 (27.9)	54 (21.6-135.6)	60-70%	0	BNT162b2	BNT162b 2	none	3	28	1 month	61

Study	Study information						Population Characteristics					Information on Vaccination						
	Country	Study design	Participants	Special Population	Intervention	Study population	Age	*N* female (%)	Transplant vintage (months)*	Triple immuno-suppressive therapy (%)	*N* with Previous SARS-CoV-2 Infection n (%)	Primary vaccination series	Third dose	Fourth dose	Total vaccine doses	Last vaccination interval (days)*	Follow up	*N* receiving additional dose
McEvoy 2022	Canada	prospective cohort study	KTRs	no	BNT162b2, mRNA-1273 **separately reported**	including seropositive patients	55.5 (45.8-63)	9 (20.5)	43.1 (7-142)	70.50 %	2 (4.5)	mRNA	mRNA	none	3	152 (127.3-185.7)	1 month	44
Midtvedt 2022	Norway	prospective cohort study	KTRs	no	mRNA-1273	including only patients with low or no seroconversion after primary series	60 (12)	79 (42)	99.6±84	86 %	0	mRNA	mRNA	mRNA	4	126 (67.9-128.1)	1 month	188
Mitchell 2022	USA	prospective cohort study	KTRs	Belatacept treated patients	mRNA or Ad26.COV2.S **not reported separately**	including seropositive patients	61.9 (52.4-68.6)	17 (68)	40.8 (25.2-105.6)	NR	0	mRNA	Ad26.COV2.S or mRNA	none	3	NR	2-4 weeks	26
Noble 2021	France	prospective cohort study	KTRs	Belatacept treated patients	BNT126b2, mRNA-1273	including seropositive patients	62 (13)	18 (31.5)	122 (81) for Belatacept group vs. 174 (346) months in the tacrolimus group	NR	NR	mRNA	mRNA	none	3	NR	1 month	57
Regele 2022	Austria	controlled clinical trail (CT)	KTRs	no	BNT162b2 + <u>co-intervention:</u> MPA hold vs. standard triple	including only patients without seroconversion after primary series	63.97 (9.16)	15 (38)	47 (32.5-90.5)	100 %	0	mRNA or viral vector	mRNA or viral vector	BNT162b2	4	136 (127-141)	1 month	39
Roch 2022	Germany	prospective cohort study	KTRs	no	Ad26.COV2.S	including only patients with low or no seroconversion after primary series	64.6 (10.33)	7 (35)	NR	75 %	NR	BNT162b2	BNT162b2	Ad26.COV2.S	4	>28 days later, not closer specified	1 month	20
Schimpf 2022	Austria	prospective cohort study	KTRs	no	Ad26.COV2.S	including seropositive patients	60.6 (no SD)	30 (40)	109 months	NR	NR	mRNA	Ad26.COV2.S	none	3	109 (range 109.0-145.0)	6 weeks	76
Schrezenmeier 2021	Germany	prospective cohort study	KTRs	no	BNT162b2, ChAdOx1 **separately reported**	including only patients without seroconversion after primary series	59.7 (13.8)	11 (44)	124.8±104.28	80 %	0	mRNA	BNT162b2 or ChAdOx1	none	3	127 ± 1	2-4 weeks	25
Schrezenmeier 2022	Germany	prospective cohort study	KTRs	no	mRNA, ChadOx1 + <u>co-intervention:</u> Reduction of MPA<br>homologous and heterologous **separately reported**	including only patients without seroconversion after primary series	59.8 (14.8)	12 (41.4)	118.8± 70.8	80 %	0	mRNA	mRNA	BNT162b2	4	59.1 ±12.6	1 month	29
Seija 2022	Uruguay	prospective cohort study	KTRs	no	BNT162b2 on BNT162b2 primay series or on	including seropositive patients	58 (45-72)	41 (37.6)	68 (32-146)	76 %	0	Coronavac 2x	BNT162b2	BNT162b2	3 and 4	30	1 month	109

	Study information					Population Characteristics						Information on Vaccination						
Study	Country	Study design	Participants	Special Population	Intervention	Study population	Age	*N* female (%)	Transplant vintage (months)*	Triple immuno-suppressive therapy (%)	*N* with Previous SARS-CoV-2 Infection n (%)	Primary vaccination series	Third dose	Fourth dose	Total vaccine doses	Last vaccination interval (days)*	Follow up	*N* receiving additional dose
					Coronavac primary series, **separately reported**													
Tauzin 2022	Canada	prospective cohort study	SOTRs,KTRs	no	mRNA	including seropositive patients	46 (range: 23-73)	12 (39)	24.48 (-4.8-275.16)	>80%	n.a.	mRNA	mRNA	none	3	111 (range 34 - 153)	1 month	27
Thomson 2022	UK	prospective cohort study	KTRs	no	BNT162b2 on BNT162b2 primary series or BNT162b2 on ChadOx1-S **separately reported**	including seropositive patients	61 (51-68) for serone gative, 60 (49-67) for seropo sitive KTRs	202 (35)	NR	17 %	0	ChadOx1-S or BNT162b2	mRNA	None/ mRNA	3 and 4	167 (145-189), 174 (156-189)	1 month	586
Werbel 2021	USA	case series	SOTRs (30 in total, 23 KTRs)	no	BNT162b2, mRNA-1273, Ad26.COV2.S **separately reported**	including seropositive patients	whole study group: (57, 44 to 62)	12 (52)	87.7± 72.3	83 %	0	mRNA	Ad26.CO V2.S or mRNA	none	3	76.2±16.3	2-3 weeks	23
Wong 2022	Australia	prospective cohort study	KTRs + KPTRs	no	BNT162b2	including seropositive patients	NR	NR	NR	>85%	0	ChadOx1-S or BNT162b2	BNT162b 2	none	3	98	1 month	23
Yahav 2022	Israel	prospective cohort study	KTRs,	no	BNT162b2	including seropositive patients	59.3 (12.4)	61 (32.1)	89.76±95.7 6	85 %	NR	BNT162b2	BNT162b 2	none	3	163.38±18. 4	1 month	190

Supplemental Table S3: Antibody Titers

	Information on Vaccination					Population Characteristics					Serologic Assessment										
Study	Primary vaccination series	Third dose	Fourth dose	Total vaccine doses	Last vaccination interval (days)*	Study population	Age	*N* female	Transplant vintage (months)*	Triple immuno-suppressive therapy	Follow-up	Definition of sero-response	Assay	De-novo response after additional dose?	*N* with additional vaccine dose	AB Titers after additional dose (median)	IQR	*N* with primary dose	AB Titers before additional dose (median)	IQR	Units
Benning 2022	mRNA or ChAdOx1	mRNA	none	3	138 (117-162)	including seropositive patients	55 (46-65)	20 (41)	97.2 (28.8-163.2)	>80%	3 weeks	Anti-RBD-IgG	bead-based multiplex assay Luminex platform (LabScreen Covid Plus, One Lambda Inc.)	including titers under seropositivity threshhold	49	12322	0-21413	NR	NR	NR	MFI
Benotmane 2021	mRNA-1273	mRNA-1273	none	3	51 (48-59)	including only patients without seroconversion after primary series	57.6 (49.6 - 66.1)	61 (38.4)	63.6 (22.8-133.12)	50 %	1 month	Anti-RBD-IgG	ARCHITECT IgG II Quant test (Abbott)	de-novo seroresponse	78	586	197.2-1920.1	NR	NR	NR	AU/mL
Benotmane 2022	mRNA-1273	mRNA-1273	mRNA-1273	4	68 (63-82)	including seropositive patients	56.6 (47-64.4)	26 (39)	73.2 (26.4-136.8)	>75%	1 month	Anti-RBD-IgG	ARCHITECT IgG II Quant test (Abbott, Abbott Park, IL, USA)	including titers under seropositivity threshhold	60	112.5	13.5-260	67	13.0	2.6-66.3	BAU/mL
Caillard 2022	mRNA	mRNA	BNT162b2 or mRNA-1273 **separately reported**	4	68 (61-74.7)	including only patients with low or no seroconversion after primary series	55.9 (47.1 - 64.2)	28 (30.4)	66 (27.6-136.8)	70 %	1 month	Anti-S-IgG	NR	including titers under seropositivity threshhold	92	145	27.6-243	92	16.4	5.9-62.3	BAU/mL
Cassaniti 2022	BNT162b2	BNT162b2	none	3	180	including seropositive patients	52.6 (47.2 -60)	22 (48.9)	51 (26-78.5)	64 %	3 weeks	Anti-S-IgG	Liaison SARS-CoV-2 trimeric, Diasorin	including titers under seropositivity threshhold	24	52.5	4.8-1178	20	4.8	4.8-85.3	BAU/mL
COViNEPH 2021	BNT162b2	BNT162b2	none	3	90	including seropositive patients	54.5 (44.5 -63)	36 (32.14)	96 (42-180)	70 %	2-3 weeks	Anti-S-IgG	LIAISON SARS-CoV2-trimeric IgG	Total response including only patients with seroconversion	60	384.5	144-837	60	213.2	81.2 - 548.6	BAU/mL

	Information on Vaccination					Population Characteristics						Serologic Assessment									
Study	Primary vaccination series	Third dose	Fourth dose	Total vaccine doses	Last vaccina-tion interval (days)*	Study population	Age	*N* female	Transplant vintage (months)*	Triple immuno-suppressive therapy	Follow-up	Definition of sero-response	Assay	De-novo response after additional dose?	*N* with additional vaccine dose	AB Titers after additional dose (median)	IQR	*N* with primary dose	AB Titers before additional dose (median)	IQR	Units
Kantauskaite 2022	mRNA	Ad26.COV2.S or ChadOx1 or BNT162b2 **not reported separately**	none	3	76 (25)	including only patients without seroconversion after primary series	59.4	60 (34.5)	46 (22-111) for seronegative vs. 92 (56-152) for seropositive	88 %	3 weeks	Anti-S1-IgG	Anti- spike-S1- IgG Anti-SARS-CoV-2-QuantiVac-ELISA (EUROIMMUN)	Total response including only patients with seroconversion	56	119	76-353	NR	NR		BAU/mL
Roch 2022	BNT162b2	BNT162b2	Ad26.COV2.S	4	>28 days later, not closer specified	including only patients with low or no seroconversion after primary series	64.6 (10.33)	7 (35)	NR	75 %	1 month	Anti-S-IgG	Elecsys Anti-SARS-CoV-2 S (Roche Diagnostics)	Total response including only patients with seroconversion	8	50	11.3-342.9	8	3.7	0.6-35.9	U/mL
Seija 2022	BNT162b2	BNT162b2	none	3	30	including seropositive patients	58 (45Ð72)	41 (37.6)	68 (32-146)	76 %	1 month	Anti-RBD-IgG	COVID-19 IgG QUANT ELISA Kit (developed by Universidad de la Republica, Institut Pasteur de Montevideo and ATGen Company)	de-novo seroresponse	6	37	18-188	NR	NR		BAU/mL
Thomson 2022	ChadOx1-S or BNT162b2	mRNA	none	3	NR	including seropositive patients	NR	202 (34)	NR	17 %	1 month	Anti-S-IgG	Abbott Architect SARS-CoV-2 IgG Quant II CMIA	including titers under seropositivity threshhold	586	295	9.1-1611	586	9.2	7.1-173	BAU/mL
Thomson 2022	ChadOx1-S or BNT162b2	mRNA	BNT162b2 or mRNA-1273, **separately reported**	4	NR	including seropositive patients	NR	202 (34)	NR	17 %	1 month	Anti-S-IgG	Abbott Architect SARS-CoV-2 IgG Quant II CMIA	including titers under seropositivity threshhold	239	437	25-2211	NR	NR		BAU/mL
Yahav 2022	BNT162b2	BNT162b2	none	3	163.38 (18.4)	including seropositive patients	59.3 (12.4)	61 (32.1)	89.76 ± 95.76	85 %	1 month	Anti-S-IgG	SARS-CoV-2 IgG II Quant (Abbott)	including titers under seropositivity threshhold	190	514.35	19.35-5474.4	190	13.8	2.6-111.55	AU/mL

*usually reported as mean±SD or median (Q1-Q3). If reported as median (range), this was specified.

Supplemental Table S4: Neutralizing antibodies against Delta

		Information on Vaccination					Population Characteristics						Serologic Assessment					
Study	Country	Primary vaccination series	Third dose	Fourth dose	Total vaccine doses	Last vaccination interval (days) *	Study population	Age	*N* female (%)	Transplant vintage (months) *	Patients receiving additional dose *N*	Triple immuno-suppressive therapy	Follow-up	Definition of serologic response	Assay	De-novo response after additional dose?	Patients analyzed *N*	Response (%)
Al Jurdi 2022	USA	mRNA	mRNA	none	3	187 (181-193)	including seropositive patients	63 (54-69)	23 (43)	43 (4-443)	51	NR	1 month	Surrogate virus neutralization test (SVNT)	GenScript cPass kit	total response	51	59
Benning 2022	Germany	mRNA or ChAdOx2	mRNA	none	3	138 (117-162)	including seropositive patients	55 (46-65)	20 (41)	97.2 (28.8-163.2)	49	>80%	3 weeks	Plaque reduction neutralization test (PRNT)	in-house brew	total response	49	59
Benotmane 2022	France	mRNA-1273	mRNA-1273	mRNA-1273	4	68 (63-82)	including seropositive patients	56.6 (47-64.4)	26 (39)	73.2 (26.4-136.8)	67	>75%	1 month	Pseudotype assay	in-house brew	total response	67	58
Kantauskaite 2022	Germany	mRNA	Ad26.COV2.S or ChadOx1 or BNT162b2, **not reported separately**	none	3	76 (25)	including only patients without seroconversion after primary series	59.4	60 (34.5)	46 (22-111) for seronegative vs. 92 (56-152) for seropositive	174	88 %	3 weeks	Plaque reduction neutralization test (PRNT)	in-house brew	de novo response	56	36
McEvoy 2022	Canada	mRNA	mRNA	none	3	152 (127.3-185.7)	including seropositive patients	55.5 (45.8-63)	9 (20.5)	43.1 (7-142)	44	70.50 %	1 month	Pseudotype assay	in-house brew	total response	44	64
Roch 2022	Germany	BNT162b2	BNT162b2	Ad26.COV2.S	4	>28 days later, not closer specified	including only patients with low or no seroconversion after primary series	64.6 (10.33)	7 (35)	NR	20	75 %	1 month	Pseudotype assay	in-house brew	total response in all seropositive KTRs	9	44
Tauzin 2022	Canada	mRNA	mRNA	none	3	111 (range 34 - 153)	including seropositive patients	46 (range: 23-73)	12 (39)	24.48 (-4,8-275,16)	27	>80%	1 month	Pseudotype assay	in-house brew	total response	27	52

* usually reported as mean±SD or median (Q1-Q3). If reported as median (range), this was specified.

Supplemental Table S5: Neutralizing antibodies against Omicron

Study	Country	Information on Vaccination					Population Characteristics						Serologic Assessment					
		Primary vaccination series	Third dose	Fourth dose	Total vaccine doses	Last vaccination interval (days) *	Study population	Age (Yrs)	*N* female	Transplant vintage (months) *	Patients receiving additional dose	Triple immunosuppressive therapy	Follow-up	Definition of serologic response	Assay	De-novo response after additional dose?	Patients analyzed *N*	Response (%)
Al Jurdi 2022	USA	mRNA	mRNA	none	3	187 (181-193)	including seropositive patients	63 (54-69)	23 (43)	43 (4-443)	51	NR	1 month	Surrogate virus neutralization test (SVNT)	GenScript cPass kit	total response	51	12
																de novo response	51	12
Benning 2022	Germany	mRNA or ChAdOx1	mRNA	none	3	138 (117-162)	including seropositive patients	55 (46-65)	20 (41)	97.2 (28.8-163.2)	49	>80%	3 weeks	Plaque reduction neutralization test (PRNT)	in-house brew	total response in all seropositive KTRs	35	43
Kantauskaite 2022	Germany	mRNA	Ad26.COV2.S or ChadOx1 or BNT162b2,) **not reported separately**	none	3	76± 25	including only patients without seroconversion after primary series	59.4	60 (34.5)	46 (22-111) for seronegative vs. 92 (56-152) for seropositive	174	88 %	3 weeks	Plaque reduction neutralization test (PRNT)	in-house brew	de novo response	56	5
Kumar 2022	Canada	mRNA-1273	mRNA-1273	none	3	90	including seropositive patients	NR	NR	NR	20	NR	1 month	Pseudotype assay	in-house brew	total response	20	25
McEvoy 2022	Canada	mRNA	mRNA	none	3	152 (127.3-185.7)	including seropositive patients	55.5 (45.8-63)	9 (20.5)	43.1 (7-142)	44	70.50 %	1 month	Pseudotype assay	in-house brew	total response	44	45
Tauzin 2022	Canada	mRNA	mRNA	none	3	111 (range 34 - 153)	including seropositive patients	46 (range 23-73)	12 (39)	24.48 (-4,8-275,16)	27	>80%	1 month	Pseudotype assay	in-house brew	total response	27	70

* usually reported as mean±SD or median (Q1-Q3). If reported as median (range), this was specified.

Supplemental Table S6: T-cell response

Study	Information on Vaccination						Population Characteristics					Serologic Assessment				
	Primary vaccination series	Third dose	Fourth dose	Total vaccine doses	Last vaccination interval*	Study population	Age	*N* female	Transplant vintage (months)*	*N* receiving additional dose	Triple immunosuppressive therapy	Follow-up	Definition of T-cell response	Assay	Patients analyzed *N*	Response (%)
BOOST-TX 2021	mRNA	mRNA or Ad26.COV2.S **separately reported**	none	3	78 (56-87) days	including only patients without seroconversion after primary series	61.2 (12.4)	82 (42)	56.16 (28.92-99)/ 49.8 (20.4-87.48) months	197	90 %	29 (28-32) days for the intervention group vs. 30 (28-33) days for the control	Quantiferon/IGRA	QuantiFERON SARS-CoV-2 assays (Qiagen)	197	9
Bruminhent 2022	Sinovac 2x	ChAdOx1-S	none	3	1-2 months	including seropositive patients	51 (42-54)	13 (42)	NR	31	NR	2 weeks	ELISpot/ T-SPOT	in house brew	31	58
Cassaniti 2022	BNT162b2 2x	BNT162b2	none	3	180 days	including seropositive patients	52.6 (47.2-60)	22 (48.9)	51 (26-78.5)	45	64 %	21 days	ELISpot/ T-SPOT	in- house brew	45	76
DIAVacc 2021	BNT162b2 2x	BNT162b2	none	3	68±1 days	including seropositive patients	57 (14.4)	18(37)	90±72	48	<50%	4 weeks	Quantiferon/IGRA	Interferon gamma (IFNg) release assay (EUROIMMUN)	35	26
Roch 2022	BNT162b2 2x	BNT162b2	Ad26.COV2.S	4	>28 days, not closer specified	including only patients with low or no seroconversion after primary series	64.6 (10.33) for seronegative and 67.5 (IQR 60.5-70.75) for seropositive KTRs	7 (35)	81 (38.5-140)	20	75 %	35 (IQR 15.25) days	FACS Analysis: CD4+ T-cells	in house brew	20	55
													FACS Analysis: CD8+ T-cells			45
Thomson 2022	ChadOx1-S 2x or BNT162b2 2x **separately reported**	BNT162b2	mRNA	4	3-4 months	including seropositive patients	58 (50-66) for seronegative and 61 (53-68) for seropositive KTRs	90 (37.6)	NR	138	17 %	38 (27-55) days	ELISpot/ T-SPOT	T-SPOT Discovery SARS-CoV-2 (Oxford Immunotec)	54	20
			none	3	NR		NR	192 (34)	NR	561	16%	35 (24-46) days			30	40
Yahav 2022	BNT162b2 2x	BNT162b2	none	3	163.38±18.4 days	including seropositive patients	56.49 (SD 13.49)	17 (32.1)	92.76±99.96	190	85 %	29 days (IQR 20-33)	Quantiferon/IGRA	Interferon gamma (IFNg) release assay (EUROIMMUN)	53	13

*usually reported as mean±SD or median (Q1-Q3). If reported as median (range), this was specified.

Supplemental Table S7: Co-interventions

		Information on Vaccination							Population Characteristics						Serologic Assessment				
Study	Study design	Primary vaccination series	Third dose	Fourth dose	Total vaccine doses	Intervention	Comparator	Last vaccination interval (days)*	Study population	Age	*N* female	Transplant vintage (months)*	Patients with additional dose *N*	Follow-up	De-novo response after additional dose?	Definition of serologic response	Assay	Response Interven-tion %	Response Compara-tor %
de Boer 2022	RCT	mRNA	mRNA	none	3	mRNA vaccine + Reduction of MPA dose	mRNA vaccine + MPA- free triple regime	202 (184-215)	including seropositive patients	72(4)	12 (38)	7 (5-11.24) or 8.5 (4.5-10.5)	22	2 months	de-novo response	Anti-RBD-IgG	SARS-CoV-2 IgG II Quant (Abbott©)	33.3	100.0
Kantauskaite 2022	observational	mRNA	Ad26.COV2.S or ChadOx1 or BNT162b2 **Not reported separately**	none	3	BNT162b2 ChadOx1, Ad26.COV2.S + Reduction of MPA dose	BNT162b2 ChadOx1, Ad26.COV2.S + MPA full dose	76 ±25	including only patients without seroconversion after primary series	63 (10)	7 (29)	30 (21–68) vs. 29 (15-45)	29	3 weeks	de-novo response	Anti-S1-IgG	Anti- spike-S1-IgG Anti-SARS-CoV-2-QuantiVac-ELISA (EUROIMMUN)	29.2	4.2
Regele 2022	NRCT	mRNA or viral vector **Not reported separately**	mRNA or viral vector **Not reported separately**	BNT162b2	4	BNT162b2+ MPA hold	BNT162b2 + MPA full dose	136 (127-141)	including only patients without seroconversion after primary series	63.97 (9.16)	15 (38)	47 (32.5-90.5)	39	1 month	de-novo response	Anti-RBD-IgG	Elecsys Anti-SARS-CoV-2 enzyme immunoassay (Roche Diagnostics)	33.3	31.8
Schrezenmeier 2022	observational	mRNA	mRNA	BNT162b2	4	BNT162b2+ MPA hold	BNT126b2 after heterologous (viral vector or mRNA) vaccination + MPA hold	59.1±12.6	including only patients without seroconversion after primary series	59.8 (14.8)	12 (41.4)	118.8 (70.8)	29	1 month	de-novo response	Anti-S-IgG	anti S- IgG (EUROIMMUN)	71.4	80.0
Yahav 2022	observational	BNT162b2	BNT162b2	none	3	BNT162b2+ Reduction or hold of MPA dose	BNT162b2+ MPA full dose	NR	including seropositive patients	NR	NR	NR	19	1 month	de-novo response	Anti-S-IgG	SARS-CoV-2 IgG II Quant (Abbott©)	53	53

* usually reported as mean±SD or median (Q1-Q3). If reported as median (range), this was specified.

Supplemental Table S8: Transplant vintage of patients with and without seroconversion

		Seropositive KTRS			Seronegative KTRs		
Study	Total vaccine doses	Seropositive KTR	TX Vintage (median)	Transplant vintage (IQR)	Seronegative KTR	Transplant vintage (median)	Transplant vintage (IQR)
Abedon 2022	3	20	6.45	3.275 - 10.275	3	4.61	1.5 - 5.55
Kantauskaite 2022	3	56	7.67	4.67 - 12.67	118	3.83	1.83 - 9.25
Seija 2022	4	15	5.67	2.83 - 12.91	26	4.10	2.83 - 10.33
Werbel 2021	3	6	9.50	4.4 - 12.4	14	4.00	2.25 - 6.75

Checklist of items to include when reporting a systematic review or meta-analysis (PRISMA)

Section/topic	#	Checklist item	Reported on page #
**TITLE**			
Title	1	Identify the report as a systematic review, meta-analysis, or both.	Title page
**ABSTRACT**			
Structured summary	2	Provide a structured summary including, as applicable: background; objectives; data sources; study eligibility criteria, participants, and interventions; study appraisal and synthesis methods; results; limitations; conclusions and implications of key findings; systematic review registration number.	First page
**INTRODUCTION**			
Rationale	3	Describe the rationale for the review in the context of what is already known.	Page 4
Objectives	4	Provide an explicit statement of questions being addressed with reference to participants, interventions, comparisons, outcomes, and study design (PICOS).	Page 4
**METHODS**			
Protocol and registration	5	Indicate if a review protocol exists, if and where it can be accessed (e.g., Web address), and, if available, provide registration information including registration number.	Page 4
Eligibility criteria	6	Specify study characteristics (e.g., PICOS, length of follow-up) and report characteristics (e.g., years considered, language, publication status) used as criteria for eligibility, giving rationale.	Pages 4 to 5
Information sources	7	Describe all information sources (e.g., databases with dates of coverage, contact with study authors to identify additional studies) in the search and date last searched.	Pages 4 to 5 and Appendix
Search	8	Present full electronic search strategy for at least one database, including any limits used, such that it could be repeated.	Appendix
Study selection	9	State the process for selecting studies (i.e., screening, eligibility, included in systematic review, and, if applicable, included in the meta-analysis).	Figure 1

Section/topic	#	Checklist item	Reported on page #
Data collection process	10	Describe method of data extraction from reports (e.g., piloted forms, independently, in duplicate) and any processes for obtaining and confirming data from investigators.	Pages 4 to 5
Data items	11	List and define all variables for which data were sought (e.g., PICOS, funding sources) and any assumptions and simplifications made.	Pages 4 to 5
Risk of bias in individual studies	12	Describe methods used for assessing risk of bias of individual studies (including specification of whether this was done at the study or outcome level), and how this information is to be used in any data synthesis.	Pages 4 to 5
Summary measures	13	State the principal summary measures (e.g., risk ratio, difference in means).	Pages 4 to 5
Synthesis of results	14	Describe the methods of handling data and combining results of studies, if done, including measures of consistency (e.g., I^2) for each meta-analysis.	Pages 4 to 5
Risk of bias across studies	15	Specify any assessment of risk of bias that may affect the cumulative evidence (e.g., publication bias, selective reporting within studies).	Page 9
Additional analyses	16	Describe methods of additional analyses (e.g., sensitivity or subgroup analyses, meta-regression), if done, indicating which were pre-specified.	Pages 4 to 5
RESULTS			
Study selection	17	Give numbers of studies screened, assessed for eligibility, and included in the review, with reasons for exclusions at each stage, ideally with a flow diagram.	Page 6, Fig.1
Study characteristics	18	For each study, present characteristics for which data were extracted (e.g., study size, PICOS, follow-up period) and provide the citations.	Appendix Table 2
Risk of bias within studies	19	Present data on risk of bias of each study and, if available, any outcome-level assessment (see Item 12).	Appendix Fig. 1
Results of individual studies	20	For all outcomes considered (benefits or harms), present, for each study: (a) simple summary data for each intervention group and (b) effect estimates and confidence intervals, ideally with a forest plot.	Pages 6 to 9, Figures 2 and 3

Section/topic	#	Checklist item	Reported on page #
Synthesis of results	21	Present results of each meta-analysis done, including confidence intervals and measures of consistency.	n.a.
Risk of bias across studies	22	Present results of any assessment of risk of bias across studies (see Item 15).	n.a.
Additional analysis	23	Give results of additional analyses, if done (e.g., sensitivity or subgroup analyses, meta-regression) (see Item 16).	Pages 8 to 9, Appendix tables 7, 8,Appendix figure 3
DISCUSSION			
Summary of evidence	24	Summarize the main findings including the strength of evidence for each main outcome; consider their relevance to key groups (e.g., health care providers, users, and policy makers).	Page 10
Limitations	25	Discuss limitations at study and outcome level (e.g., risk of bias), and at review level (e.g., incomplete retrieval of identified research, reporting bias).	Page 10
Conclusions	26	Provide a general interpretation of the results in the context of other evidence, and implications for future research.	Page 11
FUNDING			
Funding	27	Describe sources of funding for the systematic review and other support (e.g., supply of data); role of funders for the systematic review.	Page 6
